# *S*-Adenosyl-*S*-carboxymethyl-l-homocysteine: a novel cofactor found in the putative tRNA-modifying enzyme CmoA

**DOI:** 10.1107/S0907444913004939

**Published:** 2013-05-15

**Authors:** Robert T. Byrne, Fiona Whelan, Pierre Aller, Louise E. Bird, Adam Dowle, Carina M. C. Lobley, Yamini Reddivari, Joanne E. Nettleship, Raymond J. Owens, Alfred A. Antson, David G. Waterman

**Affiliations:** a York Structural Biology Laboratory, Department of Chemistry, University of York, Heslington YO10 5DD, England; b Diamond Light Source Ltd, Diamond House, Harwell Science and Innovation Campus, Didcot, Oxfordshire OX11 0DE, England; c OPPF-UK, Research Complex at Harwell, R92 Rutherford Appleton Laboratory, Didcot, Oxfordshire OX11 0FA, England; d Division of Structural Biology, Oxford University, Wellcome Trust Centre for Human Genetics, Roosevelt Drive, Oxford OX3 7BN, England; e Bioscience Technology Facility, Department of Biology, University of York, Heslington YO10 5DD, England; f STFC, Rutherford Appleton Laboratory, Didcot, Oxfordshire OX11 0FA, England

**Keywords:** SCM-SAH, *Escherichia coli*, putative tRNA-modification enzyme, cmo^5^U biosynthesis

## Abstract

Uridine at position 34 of bacterial transfer RNAs is commonly modified to uridine-5-oxyacetic acid (cmo^5^U) to increase the decoding capacity. The protein CmoA is involved in the formation of cmo^5^U and was annotated as an *S*-adenosyl-l-methionine-dependent (SAM-dependent) methyltransferase on the basis of its sequence homology to other SAM-containing enzymes. However, both the crystal structure of *Escherichia coli* CmoA at 1.73 Å resolution and mass spectrometry demonstrate that it contains a novel cofactor, *S*-adenosyl-*S*-carboxymethyl-l-homocysteine (SCM-SAH), in which the donor methyl group is substituted by a carboxy­methyl group. The carboxyl moiety forms a salt-bridge interaction with Arg199 that is conserved in a large group of CmoA-related proteins but is not conserved in other SAM-containing enzymes. This raises the possibility that a number of enzymes that have previously been annotated as SAM-dependent are in fact SCM-SAH-dependent. Indeed, inspection of electron density for one such enzyme with known X-­ray structure, PDB entry 1im8, suggests that the active site contains SCM-SAH and not SAM.

## Abstract 2

The putative methyltransferase CmoA is involved in the nucleoside modification of transfer RNA. X-ray crystallography and mass spectrometry are used to show that it contains a novel SAM derivative, *S*-adenosyl-*S*-carboxymethyl-l-homocysteine, in which the donor methyl group is replaced by a carboxymethyl group.

## 1. Introduction

Following transcription by RNA polymerase, a transfer RNA transcript is converted into a mature tRNA through processing and nucleoside modification. Processing in bacteria entails the removal of the 5′ leader and 3′ tail sequences and, if necessary, the re-synthesis of the 3′ CCA extension required for aminoacylation. Nucleoside modification is part of the maturation process that extends the physicochemical properties of tRNA by providing a wider complement of nucleosides than the canonical four introduced by RNA polymerase during transcription. Approximately 10% of nucleosides in a typical tRNA are modified, which corresponds to around seven modified nucleosides per tRNA (Jühling *et al.*, 2009 ▶). Of the 109 modified nucleosides of RNA, 93 are found in tRNA, making it the most diversely modified RNA (Cantara *et al.*, 2011 ▶). Modified nucleosides confer diverse properties upon tRNA, but they generally fine-tune the structure and stability such that it is optimized for functioning in processes such as aminoacylation and translation (Motorin & Helm, 2010 ▶).

The anticodon stem loop (ASL) is one of the most heavily modified regions of tRNA and is frequently modified at positions 34 (the wobble position) and 37 (immediately after the anticodon) (Jühling *et al.*, 2009 ▶). Modification of the ASL reduces its flexibility and shifts its structure towards the U-­turn conformation; this has been seen in crystal structures of mature tRNA, solution structures of isolated modified ASL domains and structures of the 70S ribosome in complex with tRNA and mRNA (Selmer *et al.*, 2006 ▶; Shi & Moore, 2000 ▶; Vendeix *et al.*, 2008 ▶). Because modifications impose this conformation upon the ASL, the entropic penalty otherwise associated with remodelling during translation is avoided (Agris, 2008 ▶). A number of nucleoside modifications at positions 34 and 37 also expand the decoding capacity beyond that predicted by the ‘Wobble hypothesis’ (Agris *et al.*, 2007 ▶). In bacteria, the 5-oxyuridine derivatives commonly found at position 34 fall into this category because they allow the tRNA to decode not only codons ending in A and G (as predicted by the Wobble hypothesis), but also U and C, so that a single tRNA can consequently decode all four codons within a single box of the genetic code (Nasvall *et al.*, 2004 ▶). The derivative uridine-5-oxyacetic acid (cmo^5^U) has so far been found in tRNA^Ala^, tRNA^Pro^, tRNA^Ser^ and tRNA^Val^ (Jühling *et al.*, 2009 ▶). These modifications are therefore beneficial because they not only reduce the entropic penalty associated with translation but also reduce the number of different tRNAs that a cell must produce for decoding.

**Figure 1:**
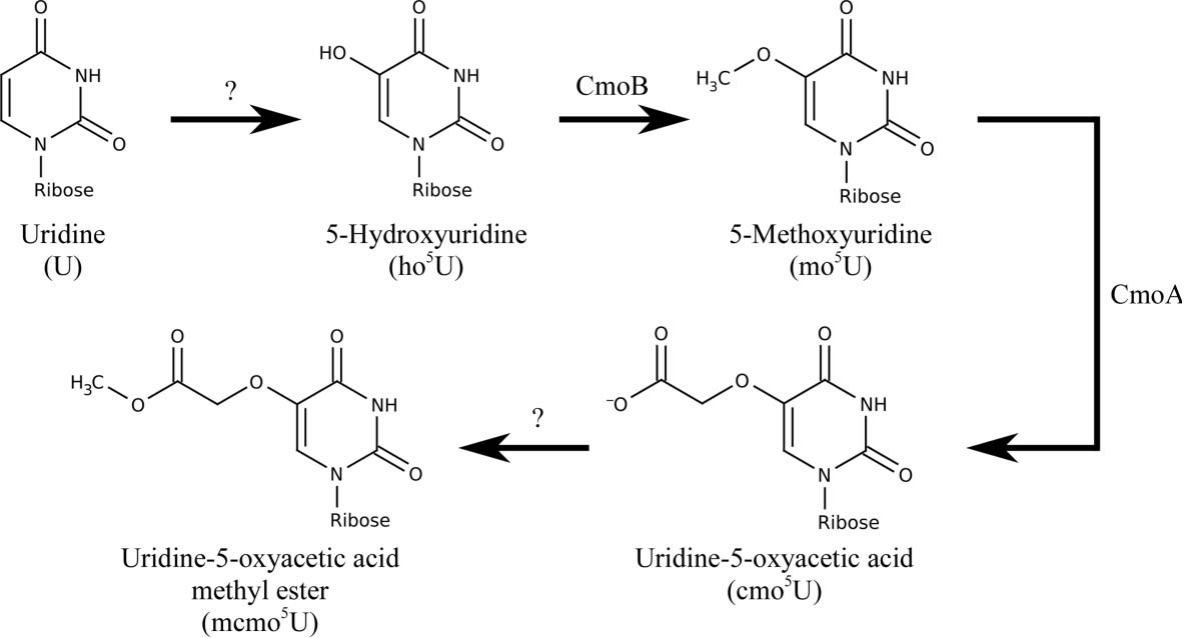
The proposed modification pathway of 5-oxyuridine derivatives. CmoA has been implicated in the modification of mo^5^U to cmo^5^U (Nasvall *et al.*, 2004 ▶), although this reaction involves more than the addition of a single methyl group, indicating that either additional enzymes and/or cofactors are involved. No enzymes involved in the conversion of U to ho^5^U or cmo^5^U to mcmo^5^U have been identified.

While the function of cmo^5^U within the context of a tRNA is well characterized, its synthesis has received relatively little attention. The proposed biosynthetic pathway involves the stepwise modification of U to cmo^5^U through the intermediates 5-hydroxyuridine (ho^5^U) and 5-methoxyuridine (mo^5^U) (Fig. 1 ▶). In keeping with the majority of other nucleoside-modification pathways, it is likely that the substrate base is modified while remaining part of the tRNA and is not simply exchanged for a pre-modified base in a transglycosyl­ation reaction. Mutations in the *cmoB* and *cmoA* genes result in accumulation of ho^5^U and mo^5^U, respectively, indicating that CmoB is involved in the modification of ho^5^U to mo^5^U and that CmoA is involved in the modification of mo^5^U to cmo^5^U (Nasvall *et al.*, 2004 ▶). Both CmoA and CmoB contain *S*-adenosylmethionine (SAM) binding motifs, hinting that they are methyltransferases, but only one of the two C atoms in the side chain of cmo^5^U is derived from SAM (Hagervall *et al.*, 1990 ▶). Furthermore, the synthesis of 5-oxy­uridine derivatives is also dependent upon chorismic acid, although the nature of this dependency has not yet been determined (Hagervall *et al.*, 1990 ▶; Nasvall *et al.*, 2004 ▶). This suggests that the modification pathway has not been fully elucidated or that parts of the cmo^5^U side chain may be derived from other metabolites.

We decided to investigate the functions of CmoA and CmoB in detail by X-ray crystallography to help fill the gaps in our understanding of cmo^5^U biosynthesis. Although CmoA was expected to be similar to the known structure of its homologue *Haemophilus influenzae* YecO, there is no sufficiently high-quality model of CmoB in the Protein Data Bank to form structure-based hypotheses about the function of the system. We hope to rectify this by providing high-quality structures of both proteins from the same target organism, allowing detailed models of the enzymatic pathway and RNA interactions to be constructed and further tested. Here, we report the structure of *Escherichia coli* CmoA, which unexpectedly reveals a cofactor that, to the best of our knowledge, has not been observed before.

## 2. Materials and methods

### 2.1. Molecular biology and protein production

The coding sequence *cmoA* was amplified from OmniMax II cells (Invitrogen) and cloned into the vector pOPINF using the In-Fusion method to generate the construct OPPF7299 (Berrow *et al.*, 2007 ▶). The final construct has an R100H point mutation with respect to the deposited sequence of CmoA from *E. coli* K-12 strain MG1655 (UniProt P76290), which may be either a PCR mutation or a genuine difference in this strain. Sequence analysis shows that amino acids with diverse properties are found at this position in other UniProt CmoA-family members. Once the X-ray structure had been determined, it became clear that this residue is located on the surface of the protein on the side opposite to the dimer interface and at a distance of ∼15 Å from the nearest atom of the SCM-SAH cofactor. *E. coli* Rosetta pLysS (DE3) cells were transformed with the resulting vector and grown in Overnight Express Instant TB medium (Merck). The cells were incubated at 310 K until an OD_600nm_ of 0.6 was attained, at which point the temperature was reduced to 298 K and the cells were grown for a further 20 h. The cells were then harvested by centrifugation and stored at 193 K.

### 2.2. Protein purification

The cells were resuspended in lysis buffer [500 m*M* NaCl, 50 m*M* Tris pH 7.5, 30 m*M* imidazole, 0.2%(*v*/*v*) Tween], lysed using a Basic Z cell disruptor (Constant Systems) and clarified by centrifugation. The supernatant was loaded onto a 1 ml HisTrap FF column (GE Healthcare) equilibrated with wash buffer (500 m*M* NaCl, 50 m*M* Tris pH 7.5, 30 m*M* imidazole) and bound protein was eluted with elution buffer (500 m*M* NaCl, 50 m*M* Tris pH 7.5, 500 m*M* imidazole). Fractions containing CmoA were concentrated and loaded onto a Superdex 200 HiLoad 16/60 column (GE Healthcare) equilibrated with gel-filtration buffer (200 m*M* NaCl, 20 m*M* Tris pH 7.5). Fractions containing CmoA were pooled and the N-­terminal hexahistidine tag was removed by digesting the protein with rhinovirus 3C protease. The mixture was then reverse-purified by performing an additional round of Ni^2+^-affinity chromatography as described above and collecting the flowthrough. This protein was then buffer-exchanged into gel-filtration buffer and concentrated to 20 mg ml^−1^ for crystallization.

### 2.3. Size-exclusion chromatography coupled with static light scattering (SEC–SLS) analysis

The oligomeric state of CmoA in solution was analysed using size-exclusion chromatography with a Superdex 200 column followed by light scattering using a Viscotek Tetra Array Detector measuring refractive index, right-angle light scattering and absorbance at 280 nm. A 100 µl sample at 0.77 mg ml^−1^ was applied onto the size-exclusion column and was observed to correspond to dimeric CmoA.

### 2.4. Crystallization

Sitting-drop experiments were performed in a CrystalQuick crystallization plate (Greiner Bio-One) at 294 K. 100 nl CmoA solution was mixed with 100 nl crystallization solution and equilibrated against a reservoir of 200 µl crystallization solution. Crystals were grown in condition E8 of the Morpheus crystallization screen (Molecular Dimensions): 0.3 *M* diethylene glycol, 0.3 *M* triethylene glycol, 0.3 *M* tetraethylene glycol, 0.3 *M* pentaethylene glycol, 0.1 *M* MOPS/HEPES-Na pH 7.5, 12.5%(*w*/*v*) PEG 1000, 12.5%(*w*/*v*) PEG 3350, 12.5%(*w*/*v*) MPD (Gorrec, 2009 ▶). Crystals grew after 5 h and were flash-cooled in liquid nitrogen without any additional cryoprotection.

### 2.5. Crystallography

Data were collected on beamline I04 of Diamond Light Source, Didcot, England and were processed with *xia*2 (Winter, 2010 ▶, Evans, 2011 ▶; Leslie, 2006 ▶; Sauter *et al.*, 2004 ▶; Zhang *et al.*, 2006 ▶). The structure was determined by molecular replacement using the structure of *H. influenzae* YecO (PDB entry 1im8; chain *B*; Lim *et al.*, 2001 ▶) with both the SAM cofactor and solvent molecules removed and the programs *CHAINSAW* (Stein, 2008 ▶) and *MOLREP* (Vagin & Teplyakov, 2010 ▶) as implemented in the *MrBUMP* pipeline (Keegan & Winn, 2007 ▶). The molecular-replacement solution contained two molecules of CmoA and had initial *R*_work_/*R*_free_ values of 45.9/45.5%. The model was then improved through alternate cycles of manual rebuilding using *Coot* (Emsley *et al.*, 2010 ▶) and restrained refinement with *REFMAC*5 (Murshudov *et al.*, 2011 ▶) using an isotropic *B* factor for each atom and one TLS group per chain (Winn *et al.*, 2001 ▶). A restraints file for *S*-­adenosyl-*S*-carboxymethyl-l-homocysteine was created using the *PRODRG*2 server (Schüttelkopf & van Aalten, 2004 ▶).

The final model contains two molecules of CmoA (residues 19–247 in chain *A* and residues 20–244 in chain *B*), two molecules of *S*-adenosyl-*S*-carboxymethyl-l-homocysteine, two molecules of MPD and 273 water molecules. Model statistics are provided in Table 1 ▶. The Ramachandran plot of the model was calculated with *RAMPAGE* (Lovell *et al.*, 2003 ▶) and the figures were created with *CCP*4*mg* (McNicholas *et al.*, 2011 ▶) and the *PoseView* server (Stierand *et al.*, 2006 ▶). The coordinates and structure factors have been deposited in the Protein Data Bank with accession code 4iwn.

### 2.6. Mass spectrometry

Samples of CmoA were purified as described above and diluted to a concentration of 5 µ*M* in 50%(*v*/*v*) aqueous acetonitrile containing 1% formic acid. These samples were introduced into the mass spectrometer using a TriVersa NanoMate ion source (Advion BioSciences) in positive-ion mode. Mass spectra were acquired using a solariX FT-MS (Bruker Daltonics) with a 9.4 T superconducting magnet. Tandem MS of the released ligand was performed by collision-induced dissociation in the hexapole (Q-CID) with argon collision gas. Spectra were processed using *DataAnalysis* v.4.0 (Bruker Daltonics). Protein mass deconvolution was performed using v.2.0 of the *SNAP* algorithm and mass measurements from released ligand and fragmentation spectra were calculated from centroided data.

### 2.7. Systematic name of the cofactor

The IUPAC name for the *S*-adenosyl-*S*-carboxymethyl-l-­homocysteine (SCM-SAH) cofactor is [(3*S*)-3-amino-3-­carboxypropyl]{[(2*S*,3*S*,4*R*,5*R*)-5-(6-aminopurin-9-yl)-3,4-di­hydroxyoxolan-2-yl]methyl}(carboxymethyl)sulfanium.

## 3. Results and discussion

The crystal structure of *E. coli* CmoA was determined by molecular replacement and was refined to *R*_work_ and *R*_free_ values of 19.6 and 23.1%, respectively, using data to a resolution of 1.73 Å (Table 1 ▶). The protein copurified with a cofactor from the *E. coli* cells that we anticipated would be either *S*-­adenosylmethionine (SAM) or *S*-adenosylhomocysteine (SAH) on the basis of the *S*-adenosylmethionine-binding motifs present in the sequence of CmoA. Unexpectedly, both molecules of CmoA contain the novel derivative *S*-adenosyl-*S*-carboxymethyl-l-homocysteine (SCM-SAH), which differs from SAM by the substitution of the methyl donor group (*R* = –CH_3_) by a carboxymethyl group (*R* = –CH_2_COOH).

**Figure 2:**
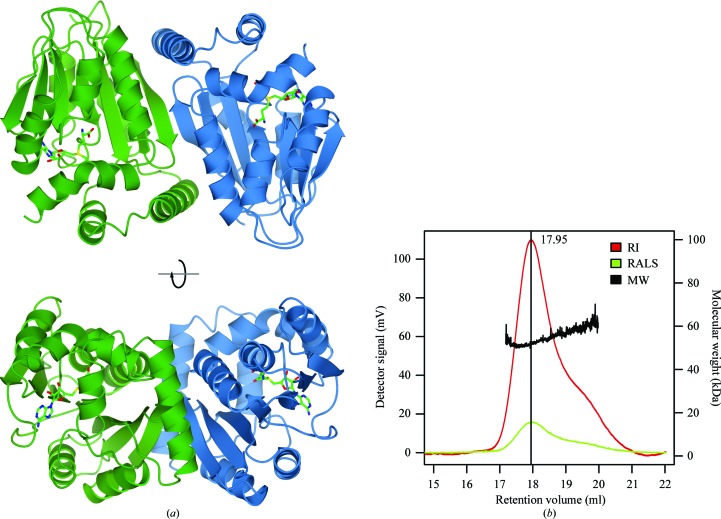
The overall structure of the CmoA dimer. (*a*) Two orthogonal views of the two monomers forming the dimer (green and blue ribbons). The *S*-adenosyl-*S*-carboxymethyl-l-homocysteine (SCM-SAH) cofactor is shown with O atoms in red, N atoms in blue and C atoms in green. (*b*) The SEC-SLS chromatogram that confirms that CmoA is a dimer in solution. The refractive index (RI; red line) and right-angle light scattering (RALS; green line) traces are displayed. The molecular weight calculated by the *OmniSEC* software (MW; black line) is shown above the elution peak. The dispersity, Mw/Mn, defined as the ratio of the weight average to number average molecular weights was reported to be 1.001, indicating a highly uniform sample.

There are two molecules of CmoA present in the asymmetric unit that are related to each other by a noncrystallographic twofold rotational axis. Apart from minor differences at the N- and C-termini, the two molecules adopt the same conformation and superpose with an r.m.s.d. of 0.3 Å (225 aligned C^α^ atoms). Analysis of the structure with *PISA* (Krissinel & Henrick, 2007 ▶) reveals that the interface between the two molecules is extensive, with 1274 Å^2^ of buried surface area per monomer (Fig. 2 ▶). In addition to an antiparallel β-­sheet formed by the β6 strands of both molecules, there are additional interactions between helix α6 of one molecule and helix α2 and strand β6 of the other molecule. Together, these interactions comprise 15 hydrogen bonds and a number of hydrophobic interactions, suggesting that the CmoA dimer present in the asymmetric unit may also represent the oligomeric state of CmoA in solution. To confirm the existence of this dimer in solution, purified CmoA was analysed by size-exclusion chromatography and static light scattering (SEC–SLS). A single species was visible on the chromatogram and the molecular weight was calculated to be 52.5 kDa, which is consistent with the theoretical molecular weight of 55.6 kDa for the dimer (Fig. 2 ▶). Interestingly, *PISA* identifies an equivalent dimer in the crystal structure of *H. influenzae* YecO (PDB entry 1im8) between chain *A* and its symmetry mate (*x* − *y*, −*y*, −*z*) with an interface area of 1194 Å^2^ per monomer (Lim *et al.*, 2001 ▶). The conserved nature of this interface indicates that the oligomeric state may be important for the structure and function of CmoA.

### 3.1. CmoA contains a novel *S*-adenosylmethionine derivative

**Figure 3:**
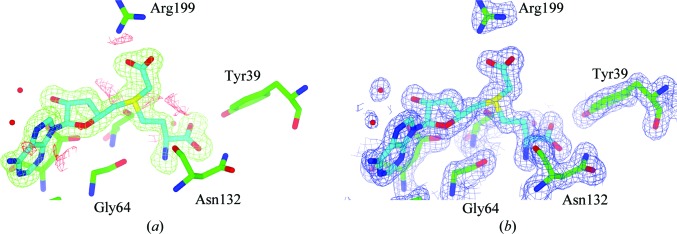
Structure of *S*-adenosyl-*S*-carboxymethyl-l-homocysteine (SCM-SAH). The final coordinates are displayed with (*a*) the likelihood-weighted *mF*_o_ − *DF*_c_ difference electron-density maps contoured at 3σ calculated prior to the modelling of SCM-SAH and (*b*) the 2*mF*_o_ − *DF*_c_ electron-density maps contoured at 1.5σ. The SCM-SAH model is shown with C atoms in cyan, O atoms in red and N atoms in blue. C atoms of protein residues are shown in green.

During the refinement of the structure, it became apparent from inspection of both the 2*mF*_o_ − *DF*_c_ and the *mF*_o_ − *DF*_c_ electron-density maps that the putative active site of CmoA contained a cofactor that was neither *S*-adenosylmethionine (SAM) nor *S*-adenosylhomocysteine (SAH). Prior to the modelling of the ligand, unambiguous positive density was visible in the *mF*_o_ − *DF*_c_ electron-density map for all of the features expected for SAM: l-methionine and both the adenine and ribose rings were visible at a contour level of 5σ, while the S atom was visible at a contour level of 18σ (Fig. 3 ▶ *a*). However, additional positive density was present at the end of the methyl group, indicating that the cofactor was a covalently modified derivative of SAM. This positive density was bifurcated, planar in shape and visible at a contour level of 7σ. Because these maps were generated prior to the modelling of any cofactor and no cofactor was present in the search model that was used during molecular replacement, the presence of this additional density was not a consequence of model bias. The shape of the density was most consistent with an *S*-­adenosylmethionine derivative in which the methyl group had been derivatized with a functional group with trigonal planar geometry.

**Figure 4:**
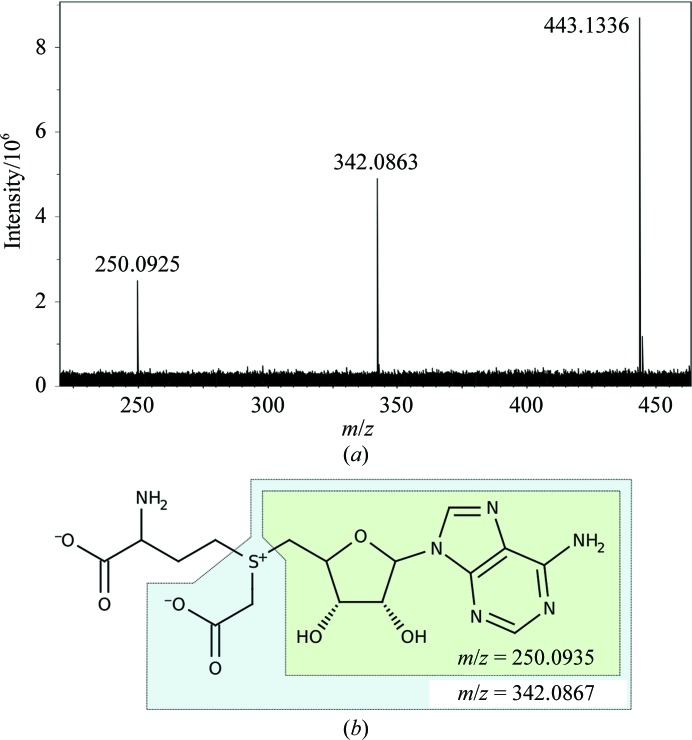
Mass spectrum of SCM-SAH. (*a*) The fragmentation spectrum and (*b*) the chemical structure of SCM-SAH. Signals consistent with the entire SCM-SAH cofactor as well as two fragments (in the blue and green boxes) were detected.

To confirm the presence of the cofactor and investigate its identity, samples of CmoA were analysed by Fourier transform mass spectrometry (FT-MS). To exclude the possibility that the modification occurred during crystallization or data collection (as a result of the chemicals present in the crystallization solution or of exposure to X-rays), the sample used for analysis was not crystallized but was from the same preparation as that used for crystallization. A signal at *m*/*z* = 27 764.2 was assigned as a protonated molecular-ion peak for CmoA, in agreement with the value of *m*/*z* = 27 763.9 estimated from the sequence alone (a difference of 0.3 Da after accounting for the proton). An additional signal at *m*/*z* = 443.1333 was detected and this was isolated and further analysed by collision-induced dissociation. The resulting fragmentation spectrum contained three signals: a parent ion at *m*/*z* = 443.1336 and two fragments at *m*/*z* = 342.0863 and 250.0925 (Fig. 4 ▶ *a*). These are not consistent with the theoretical values for SAM (*m*/*z* = 399.1445) or SAH (*m*/*z* = 385.1289), and the fragmentation spectrum featured no signals at these values. A search of the PubChem database for compounds structurally similar to SAH or SAM with a molecular weight of between 442.6 and 443.6 Da resulted in single hit: a SAM derivative in which the methyl group is substituted by a carboxymethyl group (CID 11212932; Fig. 4 ▶ *b*). We refer to this derivative as *S*-adenosyl-*S*-­carboxymethyl-l-homocysteine (or *S*-carboxy­methylated SAH), which could be further abbreviated as SCM-SAH; the full IUPAC systematic name is given in §[Sec sec2.7]2.7. The *m*/*z* values calculated for this compound are in close agreement with those determined experimentally: the parent ion has a calculated value of *m*/*z* = 443.1336 (−0.70 mDa difference) and two potential fragments may be generated with calculated values of *m*/*z* = 342.0867 and 250.0935 (−0.33 and −0.93 mDa difference, respectively; Figs. 4 ▶ *a* and 4 ▶ *b*).

Restraints for the modelling and refinement of SCM-SAH were generated with *PRODRG*2 and the ligand was then modelled into the active site of each monomer (Fig. 3 ▶ *b*). Following refinement, there was no obvious distortion of the ligand geometry with respect to the ideal geometry and there were no significant positive or negative peaks in the *mF*_o_ − *DF*_c_ difference electron-density map in the immediate vicinity of the ligand. Taken together, the crystallographic and mass-spectrometric data suggest that the active site of CmoA contains an *S*-adenosylmethionine derivative in which the methyl group is substituted by a carboxymethyl group.

### 3.2. Comparison with *H. influenzae* YecO

*H. influenzae* YecO (PDB entry 1im8) was identified as the most similar deposited structure to CmoA by both sequence-based (68% sequence identity) and structure-based (r.m.s.d. = 0.58 Å for 222 aligned C^α^ atoms) search methods (Krissinel & Henrick, 2004 ▶). The structure of YecO was originally determined by multiple-wavelength anomalous diffraction (MAD) using a selenomethionine derivative produced in *E. coli* B834 (DE3) cells grown in minimal medium in the presence of l-­selenomethionine (Lim *et al.*, 2001 ▶). The authors noted that in addition to the number of selenium sites expected on the basis of the protein sequence, one additional selenium site per monomer was detected during structure determination. This was incorporated into the cofactor that copurified with YecO, suggesting that the cofactor was derived from l-methionine (in the case of cells grown in non-labelled medium) or l-­selenomethionine (in the case of cells grown in minimal medium with l-selenomethionine). In the deposited structure of YecO the cofactor was modelled as Se-substituted SAH with a Cl^−^ ion 2.9 Å away from the Se atom.

**Figure 5:**
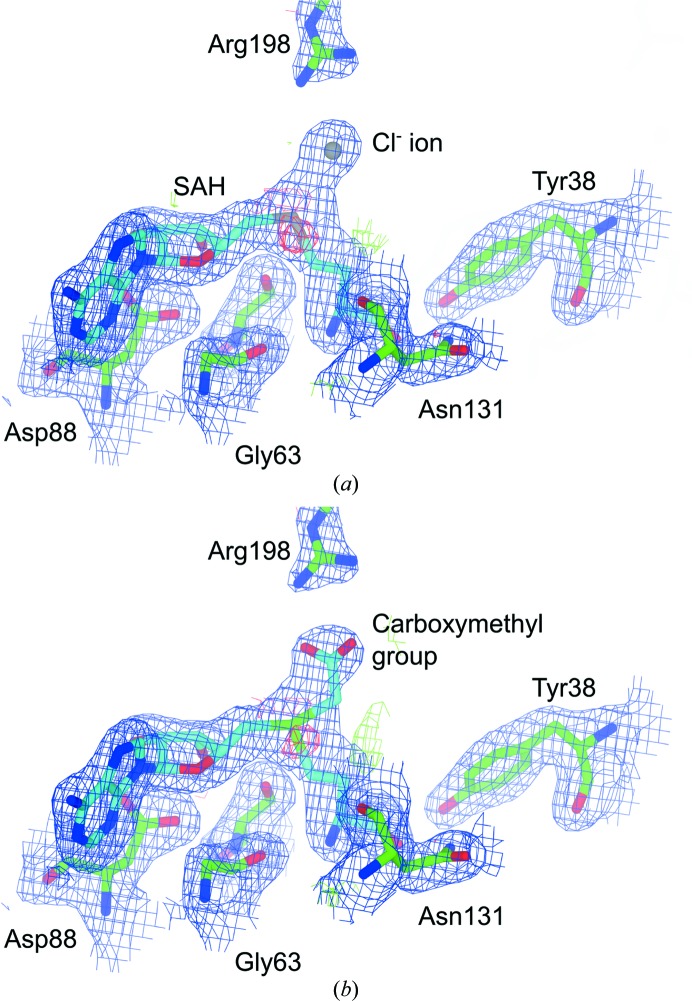
Modelling of SCM-SAH into YecO. The re-refined coordinates with either (*a*) the originally modelled Se-substituted SAH and Cl^−^ ion or (*b*) remodelled SCM-SAH are displayed along with the 2*mF*_o_ − *DF*_c_ electron-density maps contoured at 1σ and the *mF*_o_ − *DF*_c_ difference electron-density maps contoured at 3σ. SCM-SAH is depicted with C atoms in cyan, while C atoms of protein residues are shown in green.

Given the high degree of similarity between CmoA and YecO, we re-examined both the coordinates and the structure factors for YecO deposited in the PDB. Re-refinement of the deposited structure resulted in *R*_work_ and *R*_free_ values of 19.2 and 24.8%, respectively, which are comparable with the values of 18.6 and 25.5% originally reported (Fig. 5 ▶ *a*). Refinement of the YecO structure without any cofactor modelled results in electron density in both the 2*mF*_o_ − *DF*_c_ and the *mF*_o_ − *DF*_c_ electron-density maps into which the Se-substituted form of the SCM-SAH cofactor found in CmoA can be modelled, and the electron-density maps after refinement are also compatible with the presence of this cofactor (Fig. 5 ▶ *b*). However, the electron density is not defined well enough to allow a distinction between the possibilities of SAH and a Cl^−^ ion (as modelled originally) or of SCM-SAH (as modelled in CmoA). We note, however, that we were unable to find evidence of SAH and an equivalently positioned Cl^−^ ion in a manual inspection of PDB entries that are (i) annotated as methyltransferases (EC 2.1.1) and (ii) contain at least one Cl^−^ ion. Furthermore, in the case of CmoA the electron-density maps are better defined and the mass-spectrometric data argue against the cofactor modelled in YecO.

### 3.3. Overall structure of CmoA

CmoA has a Rossmann fold that comprises seven β-strands and eight α-helices. The β-strands form a single sheet in which all strands are parallel except β7. The majority of the α-helices pack against both faces of the β-sheet, although helices α2, α6 and α7 form a compact lid-like structure that sits over the region containing the SCM-SAH and renders it almost in­accessible to solvent. Superposition of CmoA with the structures of the other RNA methyltransferases TrmA (PBD entry 3bt7) and RumA (RlmD; PDB entry 2bh2) reveals that while the Rossmann-fold core is conserved between these enzymes, the lid-like structure of CmoA obstructs the region used for the binding of the RNA substrate in these methyltransferases (Lee *et al.*, 2005 ▶; Alian *et al.*, 2008 ▶). The conserved location of the substrate nucleoside with respect to the SAM cofactor in TrmA, RumA and other DNA and RNA methyltransferases suggests that the binding mode is relatively fixed. In order to place the substrate nucleoside in the corresponding position with respect to the SCM-SAH cofactor in CmoA, the lid would need to undergo a large conformational change to allow access to the cofactor and to prevent significant clashes with the neighbouring nucleotides of the tRNA substrate. An alternative possibility is that CmoA acts using additional factors which assist in the modification of the substrate uridine and does not bind tRNA directly.

**Figure 6:**
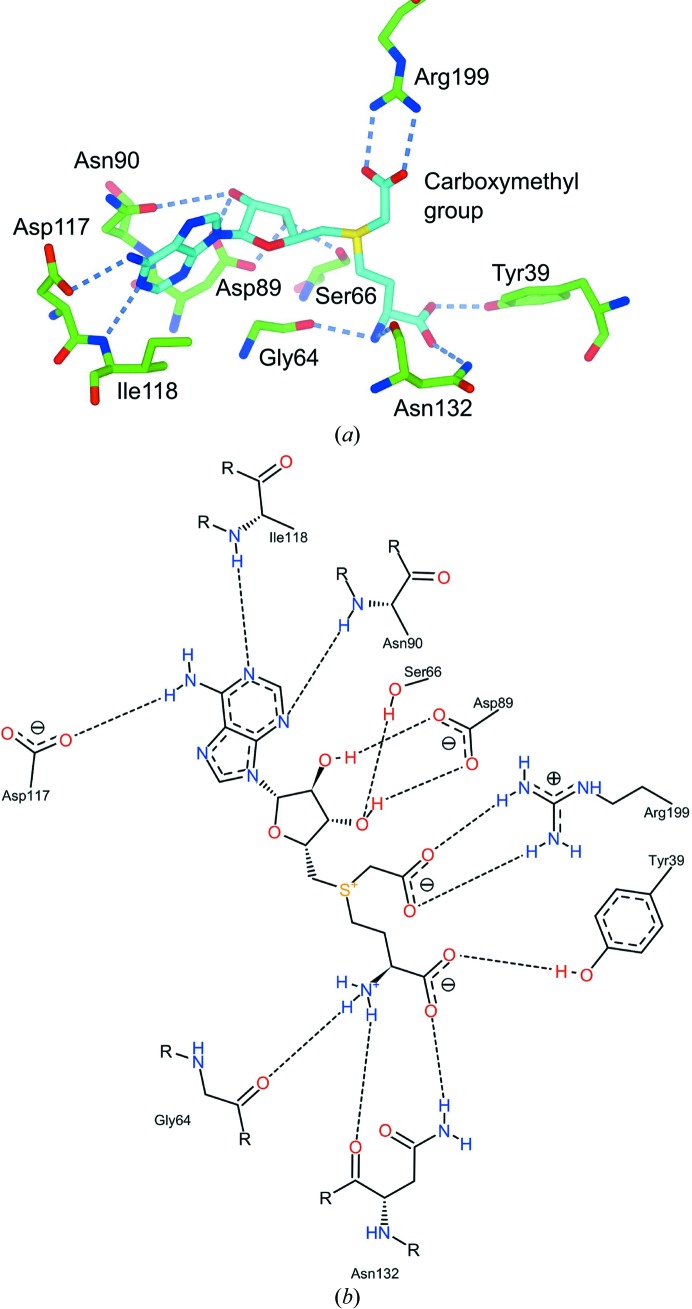
Binding of SCM-SAH by CmoA. (*a*) SCM-SAH (cyan cylinders) interacts with multiple main-chain and side-chain atoms of CmoA (green cylinders) through hydrogen bonds (blue dashes). (*b*) A two-dimensional schematic of the active site.

In common with many enzymes possessing a Rossmann fold, the majority of the conserved residues are located at the C-terminal ends of the β-strands or in the loops which immediately follow. In CmoA these residues are directly involved in contacting the SAM derivative: the adenine ring is hydrogen-bonded by the side chain of Asp117 and the main chains of Asn90, Ile118 and Phe137 (*via* a water molecule), the ribose is hydrogen bonded by the side chains of Ser66, Asp89 and Asn90 (*via* a water molecule), and the l-methionine is hydrogen bonded by the side chains of Tyr39, Asp62 (*via* a water molecule) and Asn132 and the main chains of Gly64, Ala70 (*via* a water molecule) and Asn132 (Figs. 6 ▶ *a* and 6 ▶ *b*). The negatively charged carboxylate of the carboxymethyl group interacts with the positively charged guanidinium group of Arg199 through a salt bridge with a length of 2.7 Å. The high conservation of these residues within members of the UniProt CmoA family indicates that they are important for binding the SAM derivative and may also play a role in its biochemistry.

## 4. Conclusions

We have determined the structure of *E. coli* CmoA, a putative methyltransferase that is involved in the post-transcriptional modification of U34 in a number of bacterial tRNAs. While the sequence motifs and Rossmann fold of the enzyme suggest that it is a typical SAM-dependent methyltransferase, analysis of the electron-density maps and mass-spectrometric data revealed that the protein contains an atypical SAM derivative in which the donor methyl group is replaced by a carboxy­methyl group. We name this previously unobserved derivative *S*-adenosyl-*S*-carboxymethyl-l-homocysteine (SCM-SAH). According to the UniProt database, the CmoA family currently contains 1566 proteins that are currently annotated as putative SAM-dependent methyltransferases. However, conservation of Arg199, the key residue of CmoA that stabilizes the negative charge of the carboxyl group of the SCM-SAH cofactor, suggests that these proteins contain the SCM-SAH cofactor instead of SAM and are currently annotated incorrectly. The equivalent residue in known SAM-dependent methyltransferases is not conserved. Although CmoA homologues are only found in bacteria, it is possible that such SAM derivatives are widespread in nature, being present in other enzymes currently annotated as methyltransferases.

**Figure 7:**
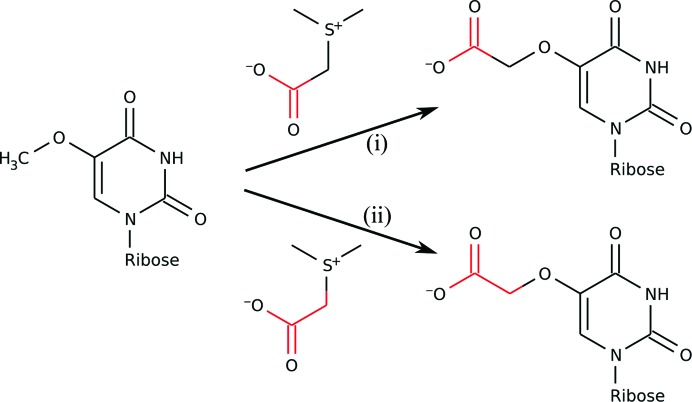
Speculative roles for SCM-SAH in the modification of mo^5^U. The modification might involve either (i) the addition of the carboxyl group (red) from SCM-SAH onto the methoxy group of mo^5^U or (ii) the substitution of the methyl group of mo^5^U with the entire carboxymethyl group.

Previous genetic studies have indicated that CmoA is involved in the modification of mo^5^U to cmo^5^U, a reaction that involves the addition of a carboxyl group onto the methoxy group of mo^5^U but that cannot be catalysed solely by a methyltransferase (Nasvall *et al.*, 2004 ▶). Assuming that the cofactor in CmoA is directly involved in modification of the tRNA, we speculate that it may participate in the formation of cmo^5^U by either (i) the transfer of just the carboxyl group of SCM-SAH onto the methoxy group of mo^5^U or (ii) the substitution of the methyl group of the side chain in mo^5^U by the entire carboxymethyl group from SCM-SAH (Fig. 7 ▶). A third possibility is that the carboxymethyl group is transferred directly onto the hydroxyl group of ho^5^U. Although this proposal is not supported by the observation that mutations in *cmoA* result in accumulation of mo^5^U and not ho^5^U, this has been suggested previously (Murao *et al.*, 1978 ▶) and a precedent for the chemistry of this reaction can be found in the O-­methyltransferases. All three possibilities, however, would be compatible with the observation that only one of the C atoms in the side chain of cmo^5^U is derived from l-methionine. The proposed mechanisms for SAM-dependent methyltransferases often involve a general base. Superpositions of CmoA with the 5-methyluridine methyltrans­ferases TrmA and RumA show that the C5 atom of the substrate uridine is neighboured by Glu164. This residue is highly conserved in members of the CmoA family and the only other amino acid found at this position is aspartic acid. As the side chain of cmo^5^U derivatives is attached to the C5 atom of the pyrimidine ring, this residue would potentially be able to act as a general base during the reaction.

Although DNA and RNA methyltransferases are able to use synthetic *S*-adenosylmethionine analogues with extended carbon chains both *in vitro* and *in vivo* (Schlenk & Dainko, 1975 ▶; Klimasauskas & Weinhold, 2007 ▶; Motorin *et al.*, 2011 ▶), there do not appear to be any reports indicating that DNA or RNA methyltransferases actually use such analogues *in vivo* for the modification of nucleic acids. We hope that our findings will lead to further characterization of the function and mechanism of CmoA and its SCM-SAH cofactor.

During the final stages of preparation of our manuscript, we became aware of PDB deposition 4gek by the New York Structural Genomics Research Consortium, in which *E. coli* CmoA is also observed in complex with SCM-SAH. A comparison of these independently determined structures adds support to the conclusions presented in this paper.

## Supplementary Material

PDB reference: CmoA, 4iwn

## Figures and Tables

**Table 1:** Data-collection and refinement statistics Values in parentheses are for the highest resolution shell.

Data collection	
Wavelength (Å)	0.9795
Space group	*P*2_1_2_1_2
Unit-cell parameters (Å)	*a* = 77.12, *b* = 91.38, *c* = 70.64
Resolution (Å)	55.9–1.73 (1.78–1.73)
No. of reflections	
Total	261435 (19274)
Unique	52750 (3856)
Completeness (%)	99.9 (100.0)
Multiplicity	5.0 (5.0)
〈*I*/σ(*I*)〉	15.1 (2.0)
*R*_merge_ †	0.056 (0.648)
*R*_p.i.m._ ‡	0.034 (0.366)
Wilson *B* factor (Å^2^)	21.6
Refinement	
Resolution (Å)	55.9–1.73 (1.78–1.73)
No. of reflections	
Working	50019 (3640)
Free	2687 (212)
No. of atoms	
Total	3942
Protein	3593
SCM-SAH	60
Solvent	289
*R*_work_ § (%)	19.6 (29.6)
*R*_free_ (%)	23.1 (31.9)
Mean *B* factor (Å^2^)	
Overall	31.1
Protein	30.7
SCM-SAH	29.6
Solvent	36.0
Geometry	
R.m.s.d., bond lengths (Å)	0.014
R.m.s.d., bond angles (°)	1.7
Ramachandran plot (%)	
Favoured	98.2
Allowed	1.8

† *R*_merge_ = 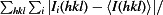 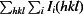.

‡ *R*_p.i.m._ = 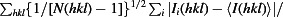 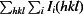.

§ *R*_work_ and *R*_free_ = 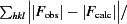 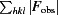. *R*_free_ was calculated from a randomly chosen set of reflections (5% of the total) excluded from the *R*_work_ set used for refinement.

## References

[bb1] Agris, P. F. (2008). *EMBO Rep.* **9**, 629–635. 10.1038/embor.2008.104 PMC2475317 18552770

[bb2] Agris, P. F., Vendeix, F. A. & Graham, W. D. (2007). *J. Mol. Biol.* **366**, 1–13. 10.1016/j.jmb.2006.11.046 17187822

[bb3] Alian, A., Lee, T. T., Griner, S. L., Stroud, R. M. & Finer-Moore, J. (2008). *Proc. Natl Acad. Sci. USA*, **105**, 6876–6881. 10.1073/pnas.0802247105 PMC2383949 18451029

[bb4] Berrow, N. S., Alderton, D., Sainsbury, S., Nettleship, J., Assenberg, R., Rahman, N., Stuart, D. I. & Owens, R. J. (2007). *Nucleic Acids Res.* **35**, e45. 10.1093/nar/gkm047 PMC1874605 17317681

[bb5] Cantara, W. A., Crain, P. F., Rozenski, J., McCloskey, J. A., Harris, K. A., Zhang, X., Vendeix, F. A., Fabris, D. & Agris, P. F. (2011). *Nucleic Acids Res.* **39**, D195–D201. 10.1093/nar/gkq1028 PMC3013656 21071406

[bb6] Emsley, P., Lohkamp, B., Scott, W. G. & Cowtan, K. (2010). *Acta Cryst.* D**66**, 486–501. 10.1107/S0907444910007493 PMC2852313 20383002

[bb7] Evans, P. R. (2011). *Acta Cryst.* D**67**, 282–292. 10.1107/S090744491003982X PMC3069743 21460446

[bb8] Gorrec, F. (2009). *J. Appl. Cryst.* **42**, 1035–1042. 10.1107/S0021889809042022 PMC3246824 22477774

[bb9] Hagervall, T. G., Jönsson, Y. H., Edmonds, C. G., McCloskey, J. A. & Björk, G. R. (1990). *J. Bacteriol.* **172**, 252–259. 10.1128/jb.172.1.252-259.1990 PMC208425 2104604

[bb10] Jühling, F., Mörl, M., Hartmann, R. K., Sprinzl, M., Stadler, P. F. & Pütz, J. (2009). *Nucleic Acids Res.* **37**, D159–D162. 10.1093/nar/gkn772 PMC2686557 18957446

[bb11] Keegan, R. M. & Winn, M. D. (2007). *Acta Cryst.* D**63**, 447–457. 10.1107/S0907444907002661 17372348

[bb12] Klimasauskas, S. & Weinhold, E. (2007). *Trends Biotechnol.* **25**, 99–104. 10.1016/j.tibtech.2007.01.006 17254657

[bb13] Krissinel, E. & Henrick, K. (2004). *Acta Cryst.* D**60**, 2256–2268. 10.1107/S0907444904026460 15572779

[bb14] Krissinel, E. & Henrick, K. (2007). *J. Mol. Biol.* **372**, 774–797. 10.1016/j.jmb.2007.05.022 17681537

[bb15] Lee, T. T., Agarwalla, S. & Stroud, R. M. (2005). *Cell*, **120**, 599–611. 10.1016/j.cell.2004.12.037 15766524

[bb16] Leslie, A. G. W. (2006). *Acta Cryst.* D**62**, 48–57. 10.1107/S0907444905039107 16369093

[bb17] Lim, K., Zhang, H., Tempczyk, A., Bonander, N., Toedt, J., Howard, A., Eisenstein, E. & Herzberg, O. (2001). *Proteins*, **45**, 397–407. 10.1002/prot.10004 11746687

[bb18] Lovell, S. C., Davis, I. W., Arendall, W. B., de Bakker, P. I., Word, J. M., Prisant, M. G., Richardson, J. S. & Richardson, D. C. (2003). *Proteins*, **50**, 437–450. 10.1002/prot.10286 12557186

[bb19] McNicholas, S., Potterton, E., Wilson, K. S. & Noble, M. E. M. (2011). *Acta Cryst.* D**67**, 386–394. 10.1107/S0907444911007281 PMC3069754 21460457

[bb20] Motorin, Y., Burhenne, J., Teimer, R., Koynov, K., Willnow, S., Weinhold, E. & Helm, M. (2011). *Nucleic Acids Res.* **39**, 1943–1952. 10.1093/nar/gkq825 PMC3061074 21037259

[bb21] Motorin, Y. & Helm, M. (2010). *Biochemistry*, **49**, 4934–4944. 10.1021/bi100408z 20459084

[bb22] Murao, K., Ishikura, H., Albani, M. & Kersten, H. (1978). *Nucleic Acids Res.* **5**, 1273–1281. 10.1093/nar/5.4.1273 PMC342075 418384

[bb23] Murshudov, G. N., Skubák, P., Lebedev, A. A., Pannu, N. S., Steiner, R. A., Nicholls, R. A., Winn, M. D., Long, F. & Vagin, A. A. (2011). *Acta Cryst.* D**67**, 355–367. 10.1107/S0907444911001314 PMC3069751 21460454

[bb24] Nasvall, S. J., Chen, P. & Bjork, G. R. (2004). *RNA*, **10**, 1662–1673. 10.1261/rna.7106404 PMC1370651 15383682

[bb25] Sauter, N. K., Grosse-Kunstleve, R. W. & Adams, P. D. (2004). *J. Appl. Cryst.* **37**, 399–409. 10.1107/S0021889804005874 PMC2808709 20090869

[bb26] Schlenk, F. & Dainko, J. L. (1975). *Biochim. Biophys. Acta*, **385**, 312–323. 10.1016/0304-4165(75)90359-1 1092359

[bb27] Schüttelkopf, A. W. & van Aalten, D. M. F. (2004). *Acta Cryst.* D**60**, 1355–1363. 10.1107/S0907444904011679 15272157

[bb28] Selmer, M., Dunham, C. M., Murphy, F. V. IV, Weixlbaumer, A., Petry, S., Kelley, A. C., Weir, J. R. & Ramakrishnan, V. (2006). *Science*, **313**, 1935–1942. 10.1126/science.1131127 16959973

[bb29] Shi, H. & Moore, P. B. (2000). *RNA*, **6**, 1091–1105. 10.1017/s1355838200000364 PMC1369984 10943889

[bb30] Stein, N. (2008). *J. Appl. Cryst.* **41**, 641–643.

[bb31] Stierand, K., Maass, P. C. & Rarey, M. (2006). *Bioinformatics*, **22**, 1710–1716. 10.1093/bioinformatics/btl150 16632493

[bb32] Vagin, A. & Teplyakov, A. (2010). *Acta Cryst.* D**66**, 22–25. 10.1107/S0907444909042589 20057045

[bb33] Vendeix, F. A., Dziergowska, A., Gustilo, E. M., Graham, W. D., Sproat, B., Malkiewicz, A. & Agris, P. F. (2008). *Biochemistry*, **47**, 6117–6129. 10.1021/bi702356j 18473483

[bb34] Winn, M. D., Isupov, M. N. & Murshudov, G. N. (2001). *Acta Cryst.* D**57**, 122–133. 10.1107/s0907444900014736 11134934

[bb35] Winter, G. (2010). *J. Appl. Cryst.* **43**, 186–190.

[bb36] Zhang, Z., Sauter, N. K., van den Bedem, H., Snell, G. & Deacon, A. M. (2006). *J. Appl. Cryst.* **39**, 112–119.

